# Prevalence, antibiotic resistance, and virulence gene profile of *Escherichia coli* strains shared between food and other sources in Africa: A systematic review

**DOI:** 10.14202/vetworld.2023.2016-2028

**Published:** 2023-10-07

**Authors:** Eustache C. Hounkpe, Philippe Sessou, Souaïbou Farougou, Georges Daube, Véronique Delcenserie, Paulin Azokpota, Nicolas Korsak

**Affiliations:** 1 Communicable Diseases Research Unit, Applied Biology Research Laboratory, Polytechnic School of Abomey-Calavi, University of Abomey-Calavi, 01 P.O Box 2009 Cotonou, Benin; 2 Department of Food Science, Faculty of Veterinary Medicine, FARAH-Veterinary Public Health, University of Liege, Quartier Vallée 2, 10 Avenue of Cureghem, Sart-Tilman, B-4000 Liege, Belgium; 3 School of Nutrition, Food Sciences, and Technology, Faculty of Agronomic Sciences, University of Abomey-Calavi, 03 P.O Box 2819, Cotonou, Benin

**Keywords:** Africa, antibiotic resistance, *Escherichia coli* virulence genes, food, systematic review

## Abstract

**Background and Aim:**

Foodborne diseases caused by *Escherichia coli* are prevalent globally. Treatment is challenging due to antibiotic resistance in bacteria, except for foodborne infections due to Shiga toxin-producing *E. coli*, for which treatment is symptomatic. Several studies have been conducted in Africa on antibiotic resistance of *E. coli* isolated from several sources. The prevalence and distribution of resistant pathogenic *E. coli* isolated from food, human, and animal sources and environmental samples and their virulence gene profiles were systematically reviewed.

**Materials and Methods:**

Bibliographic searches were performed using four databases. Research articles published between 2000 and 2022 on antibiotic susceptibility and virulence gene profile of *E. coli* isolated from food and other sources were selected.

**Results:**

In total, 64 articles were selected from 14 African countries: 45% of the studies were conducted on food, 34% on animal samples, 21% on human disease surveillance, and 13% on environmental samples. According to these studies, *E. coli* is resistant to ~50 antimicrobial agents, multidrug-resistant, and can transmit at least 37 types of virulence genes. Polymerase chain reaction was used to characterize *E. coli* and determine virulence genes.

**Conclusion:**

A significant variation in epidemiological data was noticed within countries, authors, and sources (settings). These results can be used as an updated database for monitoring *E. coli* resistance in Africa. More studies using state-of-the-art equipment are needed to determine all resistance and virulence genes in pathogenic *E. coli* isolated in Africa.

## Introduction

*Escherichia coli* is a Gram-negative intestinal bacterium that causes outbreaks of foodborne disease. Cases of recurrent *E. coli* infection have been increasing worldwide, especially in Africa, causing significant morbidity and mortality [1]. Several strains of *E. coli* are responsible for diarrheal diseases. These are diffusely adherent, such as enteropathogenic *E. coli*, enterohemorrhagic *E. coli* (EHEC), Shiga toxin-producing *E. coli* (STEC), or enterotoxigenic *E. coli* [2–6]. Different forms of *E. coli* have been isolated from the intestinal tracts of animals and humans. They are used as indicators of fecal contamination in food products or food of animal origin [7, 8]. According to the World Health Organization, 550 million people become ill and 425,000 die yearly after eating food contaminated with pathogenic microorganisms. People at risk are mainly children (especially <5 years of age)-of the 230,000 deaths recorded in Africa each year, at least 125,000 are children [9, 10]. According to a World Bank report, the financial loss caused by foodborne diseases in developing countries is estimated at US$ 95.2 billion every year, and the cost of treatment per year is estimated at US$ 15 billion [5, 11]. The World Health Organization has reported that foodborne disease outbreaks often occur in Asia and Africa, particularly in sub-Saharan Africa. Therefore, the symptomatic treatment of pathogenic *E. coli* infections is required in animals and humans [12–16].

Antibiotics are abused globally, particularly in Africa. For example, in veterinary medicine, antibiotics are used to treat animals or boost animal growth [7, 17–19]. This uncontrolled and unchecked use of antibiotics is the main cause of resistance to one or a combination of antibacterial agents [20, 21]. Several studies in Africa have reported the presence of resistance or multiresistance to antibiotics in bacteria, especially *E. coli* isolated from different matrices. In Africa, studies conducted on human and animal feces, food (meat, milk, water, vegetable or plant products, and street food), environment (soil, animal production, processing, or slaughter surfaces), or surfaces that come in contact with food during processing [21–23] have reported the presence of the same type of *E. coli* in four types of matrices, including food, water, humans, and surfaces. The virulence of *E. coli* is determined by several genes, such as *stx1*, *stx2*, *eae*, *ehly*, *ast*, *fliC07*, *rfbE0157*, *eagg*, and *papC* [21, 24, 25]. The development of this strain in several matrices poses serious public health concerns in Africa.

This study aimed to systematically review studies on the characterization, antibiotic resistance or multiresistance, virulence gene determination, and distribution of *E. coli* strains from different sources in Africa. This study showed the state of *E. coli* resistance to antibiotics in Africa through the data presented in this article. The findings of this study serve as a basis for competent authorities to make appropriate recommendations for limiting the spread of *E. coli* in Africa.

## Materials and Methods

### Ethical approval

This study does not require ethical approval.

### Study period and location

This systematic review was conducted from May 2021 to February 2023 at the University of Abomey-Calavi in Benin Republic and at the University of Liege in Belgium.

### Methodology

This systematic review of the antibiotic resistance and virulence gene profile of *E. coli* was conducted based on the PRISMA instructions [26, 27]. A data search for the literature was performed in databases such as PubMed and CAB Abstracts in addition to online search in Google Scholar and African Journal Online. The search algorithm in PubMed and CAB Abstracts was as follows: (*E. coli*) OR (Shiga-toxigenic *E. coli*)) OR (enteropathogenic *E. coli*)) OR (enterohemorrhagic *E. coli*)) OR (enterotoxigenic *E. coli*)) OR (antibiotic)) OR (resistance)) OR (virulence genes)) AND (Africa). The terms used for this algorithm were used to further search in Google Scholar and African Journal Online. All studies from 2000 to 2022 were sorted based on titles.

### Study selection and eligibility criteria

This systematic review was based on several selection criteria. Eligibility was defined according to title, year of publication, author’s origin or study area, and source of samples studied. For the title, only authors who worked on the characterization, antibiotic resistance, and determination of virulence genes of *E. coli* isolated from different sources were eligible. Articles published between 2000 and 2022 were considered. Authors of eligible articles should be of African origin or have conducted the study in Africa and on samples collected in Africa. All studies conducted on *E. coli* strains isolated from food, human, animal, environmental, and surface samples were also considered for this systematic review.

### Exclusion criteria

Some criteria were considered to exclude articles for this systematic review, such as sample size, studies not focused on antibiotic resistance and virulence genes of E.coli, studies that occurred in a matrix other than the ones in this systematic review, and studies conducted before 2000.

### Data collection

Data extracted from the articles are recorded in Table-1 [2, 3, 5–8, 10, 13–25, 28–70] . Data collected included the name of the first author, the matrix from which the bacteria were isolated, the size of samples collected for the study, methods of isolation, characterization, and determination of virulence genes of *E. coli*. Data included the year of sample collection or the year, as well as the country, in which the study was conducted, antimicrobial agents tested and resistant to bacteria, virulence genes detected, methods used for each study, and corresponding strains of *E. coli*. Epidemiological data were extracted and recorded in Excel 2016 (Microsoft Office, Microsoft Corporation, USA).

**Table-1:** Characteristics of eligible research papers.

Type of sample	Authors	Year of publication	Settings	Sample size	Methods for isolation and characterization	Type of antimicrobial resistance	Virulence genes found	Countries
Food and food products	Salamandane*et al*. [16]	2022	Street food and water	201	CT-SMAC, mPCR	Multidrug-resistant	*eaeA, stx, vt, it, astA*	Mozambic
	Madoroba*et al*. [63]	2022	Meat and meat products	2017	CT-SMAC, IMViC, rtPCR, cmPCR	not conducted	*eae, stx1, stx2, ehxA*	South Africa
	Fayemi*et al*. [6]	2021	Fresh and ready-to eat meat	180	SMAC, API 20E gallery, PCR	Multidrug-resistant	*stx1, stx2, eaeA*	Nigeria
	Alua*et al*. [30]	2021	Meat and fish	256	EMB, CT-SAMC, PCR	Multidrug resistant	*stx, hlyA, rfb0157*	Nigeria
	Geresu and Regassa [55]	2021	Minced meat, egg sandwich and cream cake	192	SMAC, EMB, RLA	Multidrug-resistant	*not conducted*	Ethiopia
	Odo*et al*. [5]	2020	Vegetables, fish, meat, soup, eggs and water	Not mentioned	SMAC, PCR	not conducted	*stx1, stx2, eaeA*	Nigeria
	Richter*et al*. [8]	2020	Fresh vegetables	545	VRBG, EMB, MALDI-TOF, PCR	Multidrug-resistant	*not conducted*	South Africa
	Adomako [31]	2020	Milk and milk products	Not mentioned	SMAC, EMB, RLA, PCR	not conducted	*stx1, stx2, eaeA, eagg, ipaH, stl*	Ghana
	Okechukwu*et al*. [32]	2020	Raw cow milk	600	EMB, GNB 24E System, PCR	Multidrug-resistant	*not conducted*	Nigeria
	Komagbe*et al*. [7]	2019	Beverage	45	API 20E, PCR	Multidrug resistant	*not conducted*	Benin
	Oje*et al*. [33]	2019	Ready-to eat foods	211	SMAC, EMB, Methyl-Red test, LA	Multidrug-resistant	*not conducted*	Nigeria
	Lupindu [3]	2018	Vegetables, fish, meat, soup, eggs and water	37	SMAC, API20E, DNA hybridization	not conducted	*stx1, stx2, eaeA*	Tanzania
	Omoruyi*et al*. [48]	2018	Beef products	60	SMAC, EMB, CHROMagar STEC, SIE test	not conducted	*alt, ast, alp*	Nigeria
	Ombarak*et al*. [10]	2016	Raw milk and cheese	172	TSB, EMB, IMViC, PCR	not conducted	*stx1, stx2, eaeA, astA, ehaA, lpfA0113, iha, hlyA, cdt, cnf*	Egypt
	Thonda*et al*. [2]	2015	Milk and milk products	Not mentioned	SMAC, EMB, RLA, PCR	Multidrug-resistant	*fliC*	Nigeria
	Abong’o and Momba [53]	2009	Meat and meat products	180	IMS, SMAC, IMViC, PCR	Multidrug-resistant	*fliCH7, rfbE0157, eaeA*	South Africa
	Beneduce*et al*. [50]	2008	Raw meat product	100	SAMC, API20E, IMS, mPCR	not conducted	*stx1, stx2, eae*	Morocco
	Benkerroum*et al*. [34]	2004	Meat product and diary	80	SAMC, IMS, PCR	not conducted	*stx1, stx2*	Morocco
Human	Amin*et al*. [57]	2022	Human stool	273	SMAC, API 20E, HBA, PCR	Multidrug-resistant	*stx1, stx2, eaeA*	Egypt
	John-Onwe*et al*. [35]	2022	Human (urine)	200	SAMC, EMB	Multidrug-resistant	*not conducted*	Nigeria
	Omebije*et al*. [36]	2021	Human feces	376	SMAC, Agglutination test	Multidrug-resistant	*not conducted*	Nigeria
	Aworh*et al*. [25]	2021	Poultry workers	122	SMAC, EMB, TSI, Microbact GNB 24E	Multidrug-resistant	*not conducted*	Nigeria
	Karama*et al*. [64]	2019	Human feces	38	LNB agar, PFGE, PCR serotyping	Multidrug-resistant	*stx1, stx2, stx2c, stx2d, eaeA, ehxA, katP, espP, etpD, saa, subA*	South Africa
	Kalule*et al*. [37]	2018	Human feces	733	CHROMagar, NHLS, TSB, SMAC,	Multidrug-resistant	*eagg, aat, eae,*	South Africa
	Too*et al*. [54]	2017	Human feces	295	SMAC, PCR, mPCR	Multidrug-resistant	*stx1, stx2, eaeA, hlyA*	Kenya
	Anago*et al*. [38]	2015	Human (stool, pus, sperm, vaginal, blood, urine)	84	API 20E, PCR	Multidrug-resistant	*not conducted*	Benin
	Raji*et al*. [60]	2008	Human stool	275	CT-SMAC, IMS, PCR	Multidrug-resistant	*stx1, stx2, eaeA*	Nigeria
	Al-Gallas*et al*. [29]	2006	Human stool	214	CT-SAMC, VCA, PCR	Multidrug-resistant	*stx1, stx2, sta, bfpA, astA, aaf/I, elt, IpaH*	Tunisa
	Olorunshola*et al*. [49]	2000	Human stool	100	SMAC, VCA, anti 0157 antisera	Multidrug-resistant	*stx1, stx2, eae, ehxA*	Nigeria
Animal	Onyeka*et al*. [67]	2020	Stool and carcass of beef	400	SMAC, RLA, mPCR	not conducted	*stx1, stx2, eaeA, hlyA*	South Africa
	Abdalla*et al*. [39]	2021	Pig	417	EMB, PCR	Multidrug-resistant	*not conducted*	South Africa
	Jaja*et al*. [19]	2020	Cattle, sheep, pigs	380	MSA, EMB, PCR	Multidrug-resistant	*not conducted*	South Africa
	Manishimwe*et al*. [40]	2021	Feces of goats, pigs, and poultry	180	SMAC, 3GCr test	Multidrug-resistant	*not conducted*	Rwanda
	Karama*et al*. [20]	2019	Cattle	140	SMAC, PCR	Multidrug-resistant	*stx2a, stx2c, stx2d, eaeA, stx1c, stx1d*	South Africa
	Montso*et al*. [51]	2019	Cattle feces	780	SMAC, mPCR	Multidrug-resistant	*stx1, stx2, eaeA, hlyA*	South Africa
	Ojo*et al*. [41]	2010	Cattle meat and feces	2133	TSB, SMAC, GNB 24E, LA, Serotyping, PCR	Multidrug-resistant	*stx1, stx2, eaeA, hlyA*	Nigeria
	Adamu*et al*. [14]	2018	Cattle feces	600	TSB, EMB, SMAC, Serotyping, PCR, EHL	Multidrug-resistant	*stx1, stx2, eae, ehlyA*	Nigeria
	Iwu*et al*. [68]	2021	Swine feces	169	SMAC, PCR,	Multidrug-resistant	*stx2*	South Africa
	Hiko*et al*. [59]	2008	Beef and goat feces	738	SMAC, O: H serotyping	Multidrug-resistant	*not detected*	Ethiopia
	Iweriebor*et al*. [18]	2015	Cattle feces	400	SMAC, TSB, PCR, Serotyping	Multidrug-resistant	*stx1, stx2*	South Africa
	Bennani*et al*. [61]	2011	Shellfish	619	VRBG, TBX, CT-SAMC, IMS, tPCR	not conducted	*stx1, stx2, eae, ehxA*	Morocco
	Jaja*et al*. [70]	2020	Cattle, sheep, pigs	400	SMAC, MSA, PCR	Multidrug-resistant	*not conducted*	South Africa
	Adenipekun*et al*. [42]	2015	Cattle feces	600	SMAC, API 20E gallery, PFGE	Multidrug-resistant	not conducted	Nigeria
	Kang’ethe*et al*. [52]	2007	Cattle, milk	370	SMAC, PCR	Tetracycline resistant	*stx1, stx2*	Kenya
	Chahed*et al*. [43]	2006	Bovine carcass	230	Rapid E. coli, mPCR, CT-SMAC, rtPCR	not conducted	*eae, stx1, stx2*	Algeria
Environmental	Pillay and Olaniran [47]	2016	Waste water	Not mentioned	Chrom colif agar, IMViC, PCR, mPCR	Multidrug-resistant	*hlyA, rfbE0157, stx1, stx2, eaeA, fliCH7*	South Africa
	Adefisoye and Okoh [71]	2016	Waste water	48	CCM, PCR	Multidrug-resistant	*eae, it, eagg, papC, ibeA, ipaH, daaE*	South Africa
	Abia*et al*. [46]	2015	Water and grab sediments	180	Colibert-18 Quanty-tray, EMB	Multidrug-resistant	not conducted	South Africa
	Malema*et al*. [24]	2018	Water	110	Colibert-18 Quanty-tray, PCR	Multidrug-resistant	*fliCH7, stx2, ibeA, ST, ipaH, eagg, eaeA*	South Africa
Food products, human, animal, environmental and surfaces	Agbagwa*et al*. [56]	2022	Poultry, waste water, soil, cloaca	40	EMB, TSI test, PCR	Multidrug-resistant	*not conducted*	Nigeria
	Ajuwon*et al*. [15]	2021	Carcass, caecum content and surfaces	415	CT-SMAC	Multidrug-resistant	*not conducted*	Nigeria
	Dougnon*et al*. [106]	2021	Surfaces, feces, and food products	81	SMAC, chromID ESBL, PCR	Multidrug-resistant	*fimH*	Benin
	Diab*et al*. [21]	2021	Human feces, Camels milk and feces	1080	SMAC, EMB, Serotyping, PCR	Multidrug-resistant	*stx1, stx2, eaeA, hlyA*	Egypt
	Ayoade*et al*. [58]	2021	Surfaces (hands, knives, floors, tables) water	147	SMAC, EMB, Serotyping, PCR	not conducted	*stx1, stx2, eaeA, hlyA*	Nigeria
	Ateba*et al*. [44]	2008	Cattle, pigs and humans stool	800	SMAC, HBA, PCR	Multidrug-resistant	*eae, hlyA*	South Africa
	Lupindu*et al*. [13]	2014	Cattle, human, soil, water	1046	SMAC, VCA, PCR	Multidrug-resistant	*stx1, eaf, bfpA, astA, eae, stx2, ehxA*	Tanzania
	Selim*et al*. [23]	2013	Food, water and clinical samples	384	EMB, SMAC, TSI, EHL, PCR, Serotyping	not conducted	*stx1, stx2, eae, hlyA*	Egypt
	Mersha*et al*. [62]	2010	Goats, sheep, water	711	CT-SAMC, IMS, PCR	not conducted	*stx1, stx2*	Ethiopia
	Ateba and Mbewe [66]	2011	Cattle, pigs, humans stool and water	140	SMAC, PCR	not conducted	*hlyA, rfbE0157, stx1, stx2, eaeA, fliCH7*	South Africa
	Sahar*et al*. [22]	2013	Food, human and animal feces, water, urine	384	TSB, EMB, IMViC, SMAC, mPCR, Serotyping	Multidrug-resistant	*stx1, stx2, eae*	Egypt
	Chigor*et al*. [17]	2010	Human, water	336	SMAC, EMB, VCA, anti 0157 antisera	Multidrug-resistant	*stx1, stx2, eae, ehxA*	Nigeria
	Ahoyo*et al*. [65]	2011	Humans and surfaces	420	Rapid E. coli, TSB, rtPCR	Multidrug-resistant	*not conducted*	Benin
	Abong’o and Momba [69]	2008	Vegetables, human	540	CT-SMAC, EMB, IMS, PCR	not conducted	*fliCH7, rfbE0157, eaeA*	South Africa
	Kaddu-Mulindwa*et al*. [45]	2001	Human and cattle stool	396	SMAC, API20E, DNA, hybridization	Not conducted	*Stx1, stx2, eae, eaf*	Uganda

IMS=Immunomagnetic separation, IMViC=Indole-methyl red Voges-Proskauer citrate, CRBA=Congo red binding assay, HBA=Hemolysis on blood agar, RLA=Rapid latex agglutination, cmPCR=Conventional multiplex PCR, rtPCR=Real-time PCR, MSA, qPCR, triplex PCR=Mannitol salt agar, CCA=Chromogenic coliform agar, SMAC=Sorbitol MacConkey agar, CT-SMAC=Cefixime potassium tellurite SMAC, VCA=Vero cells assay, TSB=Tryptic soy broth, EHL=Enterohemolysin production, PFGE=Pulsed-field gel electrophoresis, SIE=Serum indicator test, POL=Polymyxin B, CCM=Coliforms chromogenic medium, LNB agar=Luria-Bertam agar, LA=Latex agglutination

### Quality and bias assessment of eligible studies

All articles selected for this systematic review were evaluated according to a checklist provided by the Joana Briggs Institute [28]. To evaluate an article, all 10 questions on the checklist must be answered. A ”yes” answer was equivalent to 1/10. Therefore, all research papers with a minimum score of 6/10 were selected for this systematic review.

## Results

### Description of study selection and characteristics

**Figure-1:**
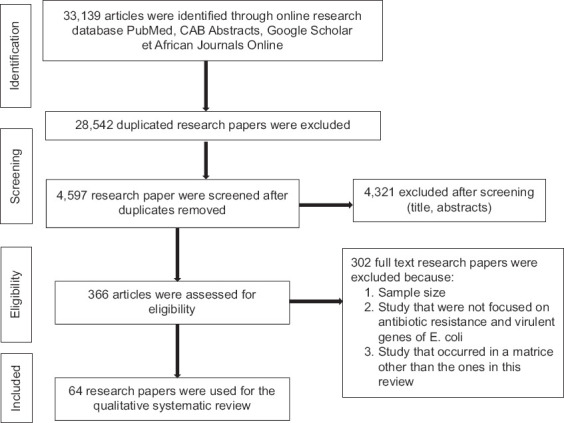
Scientific flow diagram summarizing the research process and selection of relevant studies.

**Figure-2:**
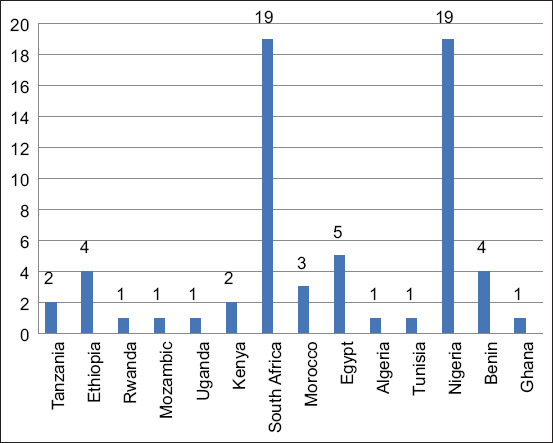
Diagram showing the number of research papers collected from each African country.

**Figure-3:**
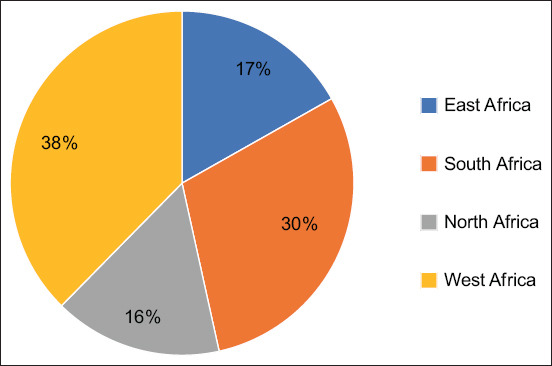
Diagram showing the percentage of research papers collected from each African zone.

For this systematic review, 33,139 articles were compiled from online databases, including Google Scholar, African Journal Online, MEDLINE (PubMed), and CAB abstracts. All articles obtained from these databases were combined, and 28,542 duplicates were removed. After evaluating the titles and abstracts of 4597 articles, 4231 articles were excluded from the study. A final analysis of 366 articles on antibiotic resistance, determination of *E. coli* virulence genes, matrices considered for the study, year, and country where the study was conducted, and sample size resulted in 64 articles from 14 African countries: 19 articles (Nigeria and South Africa); 5 articles (Egypt); 4 articles (Benin and Ethiopia); 3 articles (Morocco); 2 articles (Tanzania and Kenya); and 1 article (Ghana, Tunisia, Algeria, Uganda, Rwanda, and Mozambique) for the qualitative systematic review (Figures-1 and 2). More than 72% (n = 46) of the articles were published in the past decade (2012–2022). Almost all African regions, except Central Africa, are represented in this study. In total, 16% (n = 10) of the research papers were published in North Africa (Egypt, Morocco, Tunisia, and Algeria), 38% (n = 24) in West Africa (Nigeria, Benin, and Ghana), 30% (n = 19) in South Africa, and 17% (n = 11) in East Africa (Ethiopia, Uganda, Tanzania, Rwanda, Kenya, and Mozambique) (Figure-3). Of the 64 articles selected for this systematic review, 73% (n = 47) focused on antibiotic resistance and characterization of *E. coli* or characterization and determination of virulence genes of *E. coli*. In total, 44% (n = 28) of the studies addressed characterization, antimicrobial resistance, and virulence gene determination in *E. coli* in this study. Moreover, 45% (n = 29) of the samples collected for the studies were from food, 34% (n = 22) from livestock, 33% (n = 21) from human disease surveillance, and 13% (n = 8) from the environment. More than 28% (n = 18) of the articles reported that the samples collected were from ≥2 sources.

### Methods used for characterizing, testing antibiotic resistance susceptibility, and detecting virulence genes in *E. coli*

The isolation of *E. coli* strains from matrices was performed using Sorbitol MacConkey agar (SMAC), cefixime potassium tellurite added to SMAC (CT-SMAC), Rapid *E. coli*, eosin methylene blue, Colibert-18 Quanty-tray, chromogenic coliforms agar, and violet red bile agar, followed by biochemical tests (Gallery API 20E, IMS, IMViC, GNB 24E) Anti 0157antisera. Sorbitol MacConkey agar was the most used for isolation 63% (n = 40), followed by CT-SMAC 14% (n = 9). Rapid *E. coli* and Colibert-18 Quanty-tray were used in 3% (n = 2) of the studies, and other methods in <2% of the studies [41–44,]. For characterization and determination of virulence genes in *E. coli* strains, polymerase chain reaction (PCR) was used in 59% (n = 38) of the studies. Only 16% of the authors used multiplex PCR to determine virulence genes. Other methods, such as real-time PCR, Vero cell assay, or triplex PCR, were used for virulence gene determination [45–47]. Antibiotic resistance testing was performed using the disk diffusion method on Mueller-Hinton agar plates, following the recommendations of the Clinical Laboratory Standard Institute for antimicrobial susceptibility studies [18, 48, 49]. All methods used to characterize pathogenic *E. coli* and to determine virulence genes are listed in Table-1.

### Antibiotic susceptibility and virulence gene profile of *E. coli* strains isolated from food, human, animal, and environmental samples

Antibiotic resistance studies of bacterial agents isolated from the matrices showed that *E. coli* was resistant to 50 antibiotics [42–47]. The antibacterial agents used varied from one study to another. In 45% of the studies, *E. coli* strains isolated from food were resistant to ≥2 antibiotics, including cotrimoxazole, sulfamethoxazole, tetracycline, streptomycin, erythromycin, ampicillin, kanamycin, neomycin, chloramphenicol, ciprofloxacin, gentamicin, aztreonam, and cefotaxime [16, 22, 50–52]. In 33% of the studies, *E. coli* isolated from human samples was resistant to the same antibiotics [53–58]. The same finding was made for *E. coli* isolated from animal (34%) and environmental (13%) samples. A wide variety of virulence factors were reported in *E. coli*. In total, 73% of the studies were on determination of virulence genes, such as *stx1*, *stx2*, *rbf0157*, *eae*, *hlyA*, and *fliCH7*, which are characteristic of STEC [13, 59, 60]. Other types of virulence genes were also detected, including *daaE*, *eaf*, *katP*, *espP*, *espD*, *ipaH*, *ipfA0113*, and *eagg*, which are characteristic of enteropathogenic or EHEC [24, 43, 61]. All *E. coli* virulence genes identified in this systematic review are listed in Table-2.

**Table-2:** Distribution of virulence genes in sample and type of infection.

Virulence genes detected	Sources of isolation				

	Food	Human stool	Animal stool	Environmental sample	Type of infection
*stx1*	+	+	+	+	STEC
*stx2*	+	+	+	-	STEC
*eae*	+	+	+	+	EHEC, STEC
*Hly*	-	+	+	-	EHEC, STEC
*fliCH7*	+	+	+	+	STEC
*rbf0157*	+	+	+	-	STEC
*ast*	+	+	-	+	STEC
*aat*	-	+	-	-	STEC
*sub*	-	+	-	-	STEC
*eagg*	+	-	-	-	EHEC, EPEC
*katP*	-	+	-	-	EHEC, EPEC
*espP*	-	+	-	-	EHEC, EPEC
*etpD*	-	+	-	-	EHEC, EPEC
*vt*	+	-	-	-	STEC
*ibe*	-	-	-	+	EHEC, EPEC
*eha*	+	-	-	-	EHEC
*ipaH*	+	+	-	+	EHEC, EPEC
*st*	-	-	-	+	STEC
*ehx*	-	+	-	+	EHEC
*it*	+	-	-	-	STEC
*papC*	-	-	-	+	EHEC, EPEC
*saa*	-	+	-	-	STEC
*daaE*	-	-	-	+	EHEC, EPEC
*alt*	+	-	-	-	EHEC, EPEC
*sta*	-	+	-	-	STEC
*alp*	+	-	-	-	STEC
*ehlyA*	-	-	+	-	STEC
*ipfA0113*	+	-	-	-	EHEC, EPEC
*iha*	+	-	-	-	EHEC, EPEC
*cdt*	+	-	-	-	EHEC, EPEC
*cnf*	+	-	-	-	EHEC, EPEC
*stl*	+	-	-	-	EHEC, EPEC
*aaf/I*	-	+	-	-	EHEC, EPEC
*elt*	-	+	-	-	EHEC, EPEC
*bfp*	+	+	-	+	EHEC, EPEC
*fimH*	+	-	-	-	EHEC, EPEC
*eaf*	-	+	+	-	EHEC, EPEC

(+), detected, (−), not detected, STEC=Shiga-toxigenic *Escherichia coli*, EPEC=Enteropathogenic *Escherichia coli*, ETEC=Enterotoxigenic *Escherichia coli*, EHEC=Enterohemorrhagic *Escherichia coli*

### Transmission of *E. coli*

*Escherichia coli* is an Enterobacteria of fecal origin that is found in the intestines of humans and animals. Ruminants are the main reservoirs of *E. coli*. The transmission of *E. coli* occurs through several routes. Ruminants such as cow, sheep, and goat transmit *E. coli* through their feces into the environment following meat contamination during slaughter [62–64]. Thus, *E. coli* can be transmitted to humans after ingesting contaminated meat. An infected person can transmit the bacteria to another through the fecal-oral route following contact. Fish caught in contaminated water can transmit *E. coli* to humans [65, 66]. Humans can be infected after manipulating contaminated animals. Indeed, washing hands after handling farm animals is important because the risk of contamination is high when good hygiene practices are not observed. Meat products obtained from sheep, goat, beef, poultry, etc., can transmit the bacteria to humans [66–69]. Studies have shown that marine shellfish harbor bacteria [44] and *E. coli* is present in soil and water [5, 50, 70].

### Treatment and control of *E. coli* infection

#### Treatment of E. coli infection

*Escherichia coli* infections are often treated with antibiotics; however, STEC is treated symptomatically [13]. Antibiotics are ineffective in treating complications, such as hemolytic uremic syndrome (HUS), which is treated symptomatically [71]. Antibiotic treatment is not recommended for STEC-HUS because it increases the secretion of Shiga toxins (STX), and thus, the risk of developing HUS after the elimination of STEC [5, 13]. Other studies have shown their disagreement to the important role played by the class of antibiotic or bactericidal antibiotics,for example, the use of ciprofloxacin increase the risk for children to develop the disease. Studies in animal models have reported that azithromycin reduces STX release from STEC isolates and mortality *in vitro*. During the diarrhea phase, nephrotoxin use should be discontinued, and the dose of drugs excreted by the kidneys should be adjusted. Narcotics should be used cautiously in patients with renal failure because their metabolites can cause seizures [72, 73]. Therefore, symptomatic treatment requires hospitalization in specialized centers for managing of acute renal injuries.

#### Control of E. coli infection

Several strategies, especially the use of azithromycin, have been developed to control *E. coli* infection. Azithromycin reduces STX release (the main pathology of STEC) in patients with HUS. Because azithromycin is often not tested in susceptibility studies, prospective controlled studies must be conducted on STEC strains to assess the effect of azithromycin on the risk of developing HUS after STEC infection [71]. Several trials are underway in France and elsewhere to clarify the role of eculizumab - a humanized monoclonal antibody (immunoglobinG2/4 kappa) produced in an nonsecreting murine myeloma cell line using recombinant DNA technology - in managing STEC-induced HUS. Eculizumab is used to treat patients with life-threatening complications. Reservoir vaccination to reduce bacterial shedding has shown signs of success; however, the use of transgenic tobacco cells makes this approach questionable [49, 74–77]. Over the past 15 years, the use of substances, such as essential oils of *Pimenta racemosa*, *Syzygium aromaticum*, and *Cinnamomum zeynalicum*, as bactericides has been studied *in vitro* [13, 78]. *In vivo* studies directly on food products have shown conclusive results for the essential oil of *Cymbopogon citratus* [79]. Hygienic management of food and animal products remains the best strategy to control *E. coli* transmission. Intersectoral collaboration, by establishing a platform for exchanging information, between medical and veterinary professions, is needed to control the emergence and spread of *E. coli* [13, 80].

## Discussion

This systematic review was based on 64 articles that focused on antibiotic resistance and virulence genes of *E. coli* isolated from food and other sources. Data were extracted after screening the abstracts and full texts. This review focused on the methods used to characterize *E. coli*, the resistance developed by the bacteria against antibiotics, and the virulence genes that characterize its pathogenicity in different sources, including food, human, and environmental samples. In this study, Central Africa is not represented among the articles selected for the systematic review. This could be due to the lack of projects or logistical problems related to sample transport. Two countries are well represented: Nigeria (West Africa) and South Africa, which have published the largest number of articles on various types of samples [6, 80, 81]. Because South Africa and Nigeria are the two largest economies in Africa, they can fund research projects and acquire equipment for molecular biology studies. Furthermore, most studies were conducted on food and human surveillance diseases [5, 82]. Characterization of *E. coli* and virulence gene determination was performed using three methods-PCR, multiplex PCR, and real-time PCR [83, 84]. Polymerase chain reaction is the most widely used method for the characterization of *E. coli* and determination of virulence genes in most studies due to the low cost of thermal cyclers and reagents. Polymerase chain reaction has been indicated as the preferred technique for the determination of bacterial resistance and virulence genes [27, 85, 86]. Techniques such as microarray and whole-genome sequencing were not used in the reviewed articles for the characterization of *E. coli* and the determination of virulence genes [87], possibly due to their cost and the absence of equipment required for whole-genome sequencing in most African countries. Screening of the articles revealed that antibiotic resistance in *E. coli* isolated from food was similar to that of *E. coli* isolated from human surveillance diseases and environmental samples. The same finding has been made for virulence genes [88–91]. This implies that humans are contaminated after ingestion or handling of contaminated food. Transmission of bacteria from humans to food has been demonstrated in some studies. Some studies have shown contamination from food to humans [29, 92–95]. Other studies have shown that hospital or household wastewater discharged into the environment is an important source of transmission of *E. coli* to food and humans [17, 96–100].

Different classes of antibiotics were used for sensitivity testing of *E. coli* to antibacterial agents. In total, 50 antibiotics were tested on *E. coli* isolated from several types of samples (food, human, and environmental samples). Antibiotic resistance of bacteria depends on the type of sample and the study conducted. Screening of the articles revealed 31 virulence genes in Shiga *E. coli*, including *stx1*, *stx2*, *fliCH7*, *rfb0157*, *eae*, *hly*, and *fim*., which produce STX present in pathogenic *E. coli* isolated from matrices. Other authors have made the same observation in their studies on antimicrobial resistance and virulence genes of *E. coli* [2, 48, 81, 101–106]. The presence of the same virulence genes in pathogenic *E. coli* isolated from different matrices shows that the same bacteria are distributed across matrices and confirms that it can be transmitted from one matrix to another.

## Conclusion

This systematic review presents data on antibiotic resistance in pathogenic *E. coli* isolated from three main matrices (food, human samples, and the environment) and the virulence gene profile of *E. coli* from studies in 14 African countries. Only Central Africa is not represented in this study. This systematic review demonstrates the need for African governments to put in place a surveillance system to control the use of antibiotics in treating human and livestock diseases, especially those caused by *E. coli*. Plant-based solutions for treating foodborne diseases in general and those due to pathogenic *E. coli*, in particular, must be considered to limit the uncontrollable use of antibacterial agents, especially in breeding. To characterize pathogenic *E. coli* and determine virulence genes, PCR (classical PCR 16, real-time PCR, and multiplex PCR) was used in most studies. However, no study has reported the use of whole-genome sequencing for the determination of virulence genes, certainly because of its high cost. Given the advantages of whole-genome sequencing, African governments must develop partnerships with Western countries to facilitate the acquisition of this advanced equipment in African laboratories.

## Authors’ Contributions

ECH: Conceptualization of the study and conducted the study. PS and ECH: Database search, data extraction, and manuscript writing. SF, GD, and NK: Studied the titles and abstracts of the articles and extracted data. VD and PA: Carried out the quality assessment of each study. All authors have read, reviewed, and approved the final manuscript.
